# Challenges to Dementia Care in El Salvador: Perspectives from Geriatricians of the Salvadoran Older Adults Study

**DOI:** 10.1002/alz.093378

**Published:** 2025-01-09

**Authors:** Esmeralda Valdivieso‐Mora, Ricardo E. Mancia‐Zuniga

**Affiliations:** ^1^ Universidad Centroamericana Jose Simeon Canas, San Salvador, San Salvador El Salvador

## Abstract

**Background:**

The Salvadoran Older Adults Study is the first nation‐wide research project conducted at a university level in El Salvador (Central America). The study included semi‐structured qualitative interviews of seven geriatricians. There are only 12 geriatricians for over one million 200 thousand older adults in El Salvador. The present analysis examines their perspectives on dementia care in this country.

**Method:**

The Salvadoran Older Adults Study consisted of an explanatory mixed‐methods Quan‐Qual design that included a national survey of Salvadoran older adults and qualitative interviews. Seven geriatricians were interviewed as part of the study. Questions inquired about their educational background, professional career, main reason for referrals in their clinical practices, challenges faced by older adults in the public and private health systems.

**Result:**

All geriatricians indicated that dementia and neurocognitive disorders were the main reason for referrals in their private practice and public health institutions. They reported that lack of education about geriatric care among the population was the main challenge they face for providing services to older adults. Other challenges mentioned are categorized as expressions of ageism, even among their medical doctor colleagues.

**Conclusion:**

Geriatricians perceive their role in the public health system has been minimized. The referrals they receive are mostly of individuals with late stage dementia. They provide care mostly to patients that have been seen previously by a medical provider that “does not know what else to do with them”. Other challenges mentioned can be categorized as expressions of ageism present in the public health systems, their university‐level doctoral education and communities.

